# Pneumothorax Causing Pneumoperitoneum: Role of Surgical Intervention

**DOI:** 10.1155/2016/4146080

**Published:** 2016-08-30

**Authors:** Fernanda Duarte, Jessica Wentling, Humayun Anjum, Joseph Varon, Salim Surani

**Affiliations:** ^1^Universidad del Valle de México, Hermosillo, SON, Mexico; ^2^Christus Spohn Hospital, Memorial, Corpus Christi, TX, USA; ^3^Christus Spohn Hospital Residency Program, Corpus Christi, TX, USA; ^4^Critical Care Services, Foundation Surgical Hospital, Houston, TX, USA; ^5^The University of Texas Health Science Center at Houston, Houston, TX 77030, USA; ^6^Texas A&M University, Corpus Christi, TX 78413, USA

## Abstract

The most common cause of a pneumoperitoneum is a perforation of a hollow viscus and the treatment is an exploratory laparotomy; nevertheless, not all pneumoperitoneums are due to a perforation and not all of them need surgical intervention. We hereby present a case of pneumoperitoneum due to a diaphragmatic defect, which allowed air from a pneumothorax to escape through the diaphragmatic hernia into the abdominal cavity.

## 1. Introduction

In the majority of cases, pneumoperitoneum seen under the diaphragm on chest X-ray that is not iatrogenic is due to perforation of the hollow viscus but there are exceptions to these rules [1]. Clinical diagnosis of a nonsurgical pneumoperitoneum in time can prevent unnecessary laparoscopic surgery [2].

We hereby describe a case of patient with pneumothorax and a diaphragmatic hernia and an X-ray of the chest and CT scan showing pneumoperitoneum. We will also discuss the diagnosis and treatment of pneumoperitoneum along with a brief review of the literature.

## 2. Case Presentation

**Figure 1:**
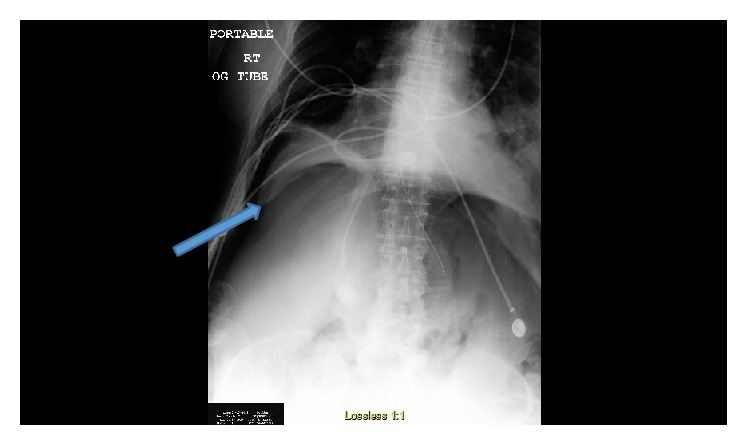
Chest/abdomen X-ray showing right pneumothorax and also air under diaphragm suggesting perforated viscus.

**Figure 2:**
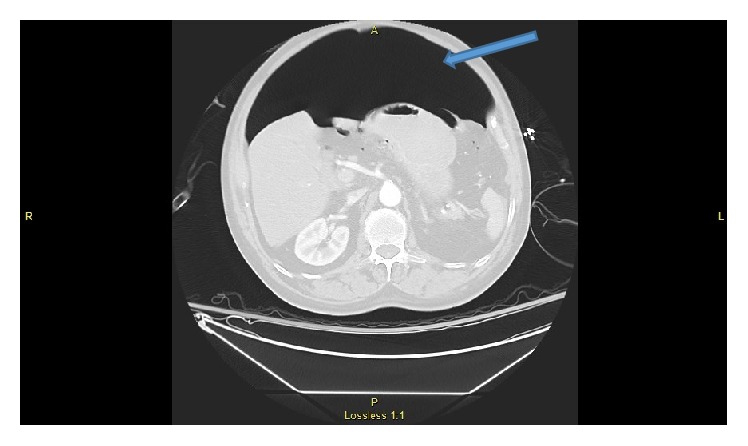
CT scan of abdomen showing large amount of air in the abdominal cavity.

**Figure 3:**
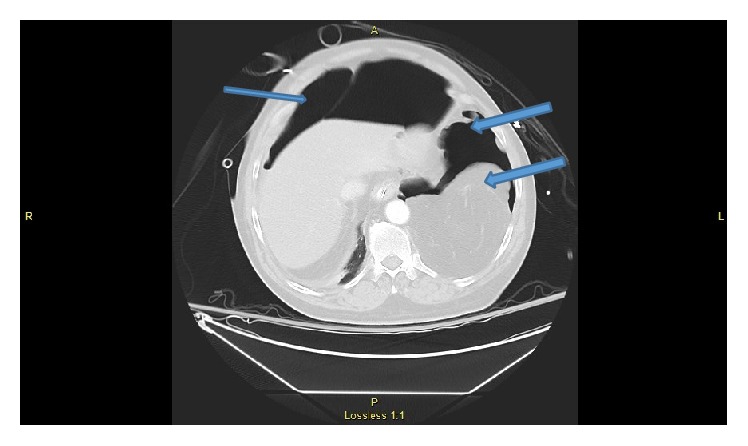
CT scan of abdomen showing pneumothorax on both sides and also herniation of omentum in left thoracic cage.

A 78-year-old gentleman with a history of asthma, chronic obstructive pulmonary disease (COPD), and hypertension presented to the Emergency Department (ED) with respiratory distress due to his chronic lung disease. Upon his arrival oxygen saturation was 80% while breathing room air; the patient was emergently placed on noninvasive mechanical ventilation utilizing bilevel positive airway pressure (BiPAP). However, shortly after his admission to the intensive care unit, he required endotracheal intubation with assisted mechanical ventilation due to worsening of his respiratory status. A postintubation chest radiograph revealed a right pneumothorax (see Figure 1). A computed tomography (CT) scan revealed a left pneumothorax with massive pneumoperitoneum and a left diaphragmatic hernia containing large amount of omentum in the left chest (see Figures 2 and 3). The patient underwent emergent bilateral tube thoracotomies. He was then taken to the operating room for an exploratory laparotomy. During such procedure, a large diaphragmatic hernia was noted with omentum going up into it requiring excision. The hernia defect was relatively small, at about 3 cm. This was repaired. Further examination did not reveal any form of bowel or gastric perforation. The patient responded well to the therapy and was eventually extubated and discharged. The patient seems to have developed spontaneous pneumothorax due to his underlying COPD and bullous lung disease. Pneumoperitoneum was found to be due to escape of air from thoracic cavity into abdominal cavity via the diaphragmatic hernia.

## 3. Discussion

Our case is interesting as our patient had no evident bowel perforation in the presence of bilateral pneumothoraxes, a diaphragmatic hernia, and massive pneumoperitoneum. He seemed to have developed spontaneous pneumothoraces due to his underlying bullae and COPD. Pneumoperitoneum was found to be due to escape of air from thoracic cavity into abdominal cavity via the diaphragmatic hernia.

In 90–95% of cases of pneumoperitoneum perforation throughout the gastrointestinal tract is usually found [3]. In most instances, this represents a serious intra-abdominal catastrophe that requires immediate surgical management [4]. However, the other 5–10% of cases that suggest free air in the peritoneal cavity can be related to gynecologic, thoracic, abdominal, postoperative, nonsurgical, or idiopathic causes [3]. With the laparoscopic surgeries being performed on a daily basis, we do see the air in abdomen, and in patients with the diaphragmatic hernia it can be an incidental finding. For example, one of us has previously reported the presence of pneumoperitoneum due to vaginal insufflation that did not require surgical intervention [5]. Other cases of nonsurgical pneumoperitoneum have been reported after cardiopulmonary resuscitation and tracheal ruptures due to endotracheal intubation and most commonly due to mechanical ventilation. When talking about mechanical ventilation being a cause of pneumoperitoneum, an incidence of up to 7% of all ventilated patients has been reported; however no new studies have assessed this percentage since 1986 [6, 7].

In our present case, pneumoperitoneum neither was due to a perforation nor was postoperative but was due to potential pathway that allowed air into the peritoneum through a diaphragmatic hernia defect [8]. This has been described in some infants that undergo repair of congenital diaphragmatic hernias, in which the most common factor that correlates with death is perioperative pneumothorax due to air leakage provided by supplemental oxygen [9, 10].

A simple X-ray has a sensitivity of 74% and a CT of 92% in demonstrating free intraperitoneal gas. When there is a perforation of either the stomach, duodenum, or colon a CT with contrast is 100% sensitive and the diagnosis is fairly simple; however it is uncertain when it involves small bowel perforations [11].

Once the diagnosis of pneumoperitoneum is stated, the next step is to treat the cause; surgery is indicated if contrast and/or free intra-abdominal fluid is found in the abdomen, not only due to the presence of pneumoperitoneum. If the only radiological sign is pneumoperitoneum, it is important to admit the patient and keep them in close observation with repeated evaluation [12].

Our patient had high positive pressures during mechanical ventilation as well as an exacerbation of his COPD that led to bilateral pneumothorax and the hernia defect allowed this air to leak into the abdominal cavity and cause the pneumoperitoneum. In some cases conservative care could have been optimal, as placement of bilateral chest tubes to evacuate the pneumothoraces. In this case surgical intervention was necessary in order to repair the hernia defect to avoid future complications in a patient who was unstable on mechanical ventilation.

## 4. Conclusion

Pneumoperitoneum can present due to a diaphragmatic defect, which allows air from a pneumothorax to escape through the diaphragmatic hernia into the abdominal cavity. In these cases, not every patient requires abdominal surgical intervention, especially if the patient did not present with acute abdomen. When diagnosing pneumoperitoneum the most important part of treatment is to identify the cause, to properly treat the patient.

Keeping in mind that our patient did not have a bowel perforation, treating the pneumothorax could have been enough treatment.

It is important to stay alert for possible causes of nonsurgical pneumoperitoneum to avoid unnecessary surgical procedures.
